# Exploring Evidence-Based Approaches to Ocular Allergy Among Australian Health Practitioners

**DOI:** 10.3390/jcm15010015

**Published:** 2025-12-19

**Authors:** Ereeny Mikhail, Mohammadreza Mohebbi, Serap Azizoglu, Khyber Alam, Cenk Suphioglu, Moneisha Gokhale

**Affiliations:** 1 NeuroAllergy Research Laboratory (NARL), School of Life and Environmental Sciences, Deakin University, Waurn Ponds, Geelong, VIC 3216, Australia; 2 Deakin Optometry, School of Medicine, Deakin University, Waurn Ponds, Geelong, VIC 3216, Australia; serap.azizoglu@deakin.edu.au (S.A.); moneisha.gokhale@deakin.edu.au (M.G.); 3 Institute for Mental and Physical Health and Clinical Translation (IMPACT), Deakin University, Waurn Ponds, Geelong, VIC 3216, Australia; 4 Biostatistics Unit, Faculty of Health, Deakin University, Burwood, VIC 3125, Australia; 5 Health and Medical Sciences, Department of Optometry, University of Western Australia, Crawley, WA 6009, Australia

**Keywords:** ocular allergy, health practitioners, evidence-based practices

## Abstract

**Background/Objectives**: Ocular Allergy (OA) has profound effects on the quality of life (QoL) and ocular health of affected individuals. This study aimed to survey health practitioners in Australia on their knowledge and practices regarding currently available evidence-based diagnostic, treatment, and collaborative care approaches to OA. **Methods**: The Survey on Ocular Allergy for Health Practitioners (SOAHP), a validated tool, was distributed to various health practitioners across Australia in 2022. The survey data were analysed using descriptive statistics, Fisher’s exact test, and non-parametric tests. **Results**: A total of 155 participants completed the survey including Allergists/Immunologists (n = 6), General Practitioners (GPs) (n = 29), Ophthalmologists (n = 11), Optometrists (n = 66) and Pharmacists (n = 43). The survey revealed strengths and weakness in health practitioner approaches to OA. In terms of diagnosis, a significant 83.2% of participants were aware that itchy eyes are the hallmark symptom of OA; however, only 67.7% were aware that histamine is what causes the itching. Further to this, 57.4% of participants did not ask about QoL in clinical practice. In terms of management, only 30.3% were aware that some topical allergy eye drops act on eosinophils, and 74.9% were aware of the indications of mast cell stabiliser use. Finally, in terms of collaborative care, 68.4% did not believe there was a clear collaborative care model in Australia. **Conclusions**: This study revealed patterns in health practitioner approaches to OA. As expected, Ophthalmologists and Optometrists exhibited higher awareness and implementation of evidence-based approaches, compared to GPs and Pharmacists. However, these distinct patterns are likely influenced by differences in training and clinical responsibilities. Nonetheless, all practitioner groups showed gaps in knowledge and evidence-based practices surrounding OA. Thus, educational initiatives are required to ensure best patient-centered care is achieved, with reduced burden on the healthcare system.

## 1. Introduction

Research surrounding Ocular Allergy (OA) has grown significantly over the last 50 years, yet disparities remain among health practitioners in the diagnosis, treatment, and collaborative care approaches to OA [1,2,3].

For instance, there is a lack of consensus amongst health practitioners in the diagnosis of OA. A study involving Ophthalmologists and General Practitioners (GPs) from nine countries in Eastern Europe and the Middle East examined the differential diagnosis of red eye, and found that OA, specifically allergic conjunctivitis, was the most common ocular diagnosis during allergy season [1]. However, the agreement in the diagnosis between Ophthalmologists and GPs was only 48% [1]. This reveals a potential lack of knowledge regarding diagnostic features of OA.

Inadequate treatment of OA by health practitioners is another challenge [2,3]. A survey conducted in Europe explored the burden of allergic rhinitis in patients with both nasal and ocular symptoms who were receiving treatment [2]. It revealed that practitioners often underestimated the severity of the condition compared to patient reports [2]. This discrepancy and lack of consideration for quality of life (QoL) may lead patients to seek unnecessary over-the-counter (OTC) medications for self-treatment [4] potentially contributing to long-term negative health outcomes. Similar findings were reported in the United States of America (USA), where many patients’ allergic rhinitis needs remained unmet [3].

Despite the need for a collaborative care model for OA, the current literature lacks a comprehensive approach with potential global clinical implications. There is a clear gap in how health practitioners diagnose and treat OA, yet the specific gaps in the knowledge and practices remain unclear. Therefore, the aim of this research article is to explore the knowledge and practice patterns of health practitioners regarding OA in Australia. This is necessary as previous research in Australia has revealed that there is an ‘evidence-to-practice gap’ in other ocular conditions [5]. Therefore, conducting a survey on OA evidence-to-practice among health practitioners is timely.

## 2. Materials and Methods

### 2.1. Survey

The development and validation of the Survey on Ocular Allergy for Health Practitioners (SOAHP) [6] followed a structured five-step process: item extraction, face and content validity, pilot study, test–retest reliability, and finalisation. Initially, items were extracted from a comprehensive literature review covering diagnosis, treatment, and collaborative care of OA, leading to the formulation of various domains [6]. Face and content validity were assessed by 15 experts from relevant health fields using the modified Delphi technique, ensuring each item’s relevance, essentiality, and clarity [6]. A pilot study was conducted with 15 new participants across five specialties to evaluate comprehension and address potential response biases [6]. Subsequently, the test–retest reliability was evaluated using a sample of 25 practitioners, who were not involved in the earlier content validity and pilot phases, to complete the survey twice within one to two weeks. Reliability was analysed through various metrics, including percentage agreement and intraclass correlation coefficients (ICC) [6]. Following these assessments, the SOAHP was finalised as a smart survey with adaptive questioning, ready for wider administration to qualified health practitioners registered with the Australian Health Practitioner Regulation Agency (AHPRA) [6].

For this study, the survey was administered via Qualtrics, Provo UT in 2022, adhering to the Declaration of Helsinki and receiving human ethics approval from Deakin University (reference number: SEBE-2020-68-MOD01), with participant consent ensured through a plain language statement and consent form.

### 2.2. Participants

#### 2.2.1. Inclusion and Exclusion Criteria

SOAHP was administered to qualified health practitioners including Allergists/Immunologists, GPs, Ophthalmologists, Optometrists, and Pharmacists. The health practitioners needed to be AHPRA registered and practicing in Australia. Participants with any level of experience was appropriate as the aim was to gain a general overview on knowledge and practices surrounding OA.

However, individuals who were not fully qualified such as students, interns, residents, registrars, and/or in training were excluded from participation, as were practitioners not listed above such as Dermatologists, to maintain the focus of the study on the specified specialties.

#### 2.2.2. Sample Size and Recruitment

A sample size calculation for estimating the proportion of correct responses based on plausible scenarios about the distribution of survey items was calculated. It was assumed that the prevalence of correct response ranged between 20% and 40%. The sample size aimed to estimate the prevalence of correct responses with an absolute precision of ±0.075 (7.5%) in estimating the prevalence. Based on these assumptions, a minimum sample size of 28 to 41 for each specialty was needed.

Participants were recruited through multiple avenues including advocacy organisations, social media, and direct email. The advocacy organisations involved in participant recruitment included the Royal Australian College of General Practitioners (RACGP), Australian Medical Association (AMA), MiVision, Australasian Society of Clinical Immunology and Allergy (ASCIA), and Australian and New Zealand Society for Immunology (ASI).

### 2.3. Statistical Methods

Survey results were analysed using descriptive statistics, Fisher’s exact test, and non-parametric tests. Descriptive statistics were used for summarising questions that included multiple response selections, Fisher’s exact test was employed for questions with nominal responses, and a non-parametric test was the chosen method for questions on a Likert scale. Statistics were performed on STATA Version 17.0 software.

## 3. Results

### 3.1. Characteristics

A total of 155 health practitioners participated in the study, including six Allergists/Immunologists, twenty-nine GPs, eleven Ophthalmologists, sixty-six Optometrists, and forty-three Pharmacists. Participant characteristics are described in Table 1. The sample included a higher number of females (62.6%) than males (37.4%), with a mean age of 36.2 ± 12.0 years, and mean years of practice being 10.0 ± 10.1. Participants were distributed across all Australian states (excluding Australian Capital Territory and Northern Territory) and practiced in diverse settings under different modalities of practice. The data was collected over a period of 4 months (between June and September 2022). The average time taken to complete the survey was 27.5 ± 21.8 min, excluding 13 outliers who exceeded 150 min (these participants may have paused the survey and resumed at their convenience). The distribution of demographics by profession is represented in Table 2.

Most health practitioners self-reported that they saw OA patients on a weekly basis (33.5%), followed by monthly (25.2%), then occasionally (18.7%), then daily (14.8%), then rarely (6.5%), and finally, never (1.3%). The highest rate of seeing patients daily was with Optometrists at 21.2% and the lowest rate of seeing patients daily was with Allergists/Immunologists at 0%. Nonetheless, Allergists/Immunologists had the highest rate of seeing patients on a weekly basis at 50%. Furthermore, the highest rate of never seeing patients was with Pharmacists at 4.7%, whereas all other health practitioners did not select ‘never’ as an option.

**Figure 1:**
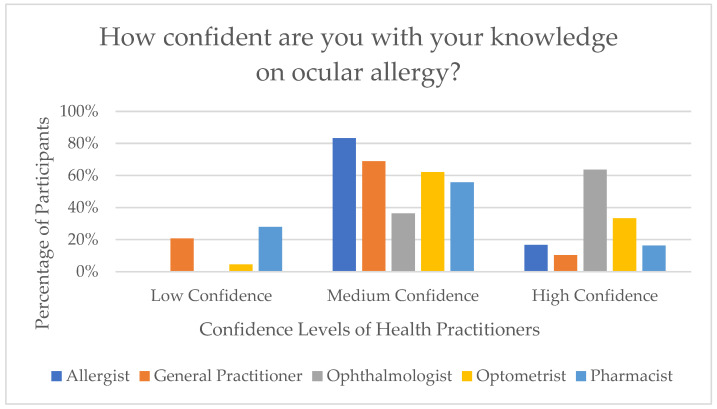
Knowledge confidence on ocular allergy by health practitioner.

Moreover, participants were asked to rate their confidence in their knowledge of OA. The majority (60.6%) reported medium confidence, followed by 25.8% who reported high confidence, and 13.5% who indicated low confidence. Ophthalmologists demonstrated the highest level of confidence, with 63.6% reporting high confidence and none reporting low confidence. In contrast, Pharmacists and GPs reported the lowest confidence, with 27.9% and 20.7% indicating low confidence, respectively. Figure 1 demonstrates the distribution of each health practitioner’s knowledge confidence.

Finally, in response to a survey question on the frequency of receiving education on OA, the majority of participants reported receiving education occasionally (41.3%) or rarely (40.0%), followed by never (9.0%), monthly (7.1%), and weekly (2.6%). Ophthalmologists had the highest rates of weekly education at 9.1%, followed by Optometrists at 4.6%. Conversely, 20.9% of Pharmacists and 17.2% of GPs selected ‘never’, showing the lowest rates of receiving education.

### 3.2. Quality of Life in Ocular Allergy Patients

Participants were asked on whether they inquire about QoL in their OA patients. Overall, 42.6% of participants responded ‘Yes’ while 57.4% responded ‘No’. A comparison by profession using Fisher’s exact test (*p* = 0.024) was statistically significant. Allergists/Immunologists had the highest rates of asking about QoL at 66.7%, whereas Pharmacists and GPs had the lowest rates at 25.6% and 34.5%, respectively. Interestingly, Ophthalmologists (54.4%) and Optometrists (53.0%) had nearly identical rates of asking patients about QoL. This is presented in Table 3.

Furthermore, participants who reported asking about QoL in their OA patients were further queried on how they implemented this practice. The options provided included asking their own QoL questions or using standardised questionnaires. The majority (n = 63, 92.6%) of participants indicated they asked their own QoL questions. However, only three participants reported using the validated Eye Allergy Patient Impact Questionnaire (EAPIQ) [7], and two participants selected ‘other’ methods for assessing QoL.

**Figure 2:**
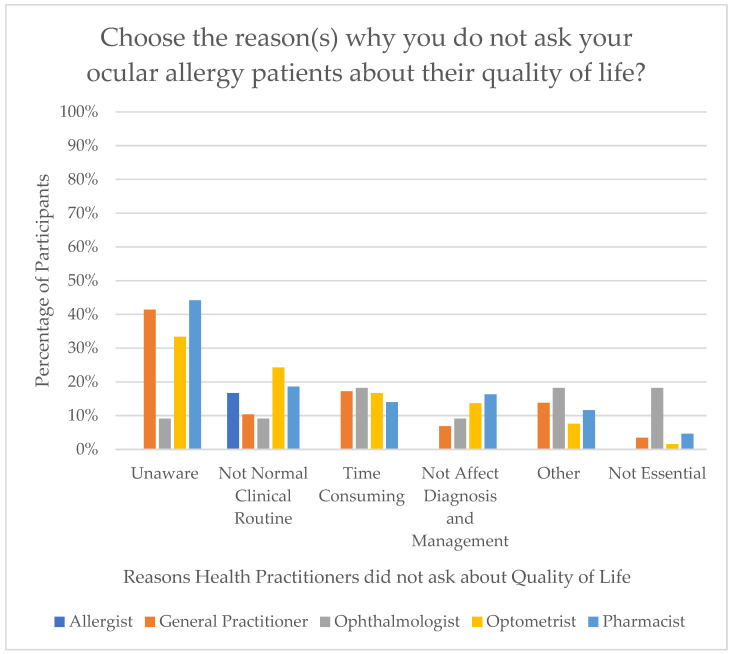
Reasons for not asking about quality of life by health practitioner.

For participants who indicated they do not assess QoL in their OA patients, the reasons for this were explored. The most commonly cited reason was a lack of awareness regarding available questionnaires (n = 54). This was followed by QoL not being integrated into their clinical routine (n = 29), the perceived time-consuming nature of QoL assessments (n = 24), the belief that it would not influence diagnosis or management (n = 19), other miscellaneous reasons (n = 16), and lastly, the view that assessing QoL is not essential (n = 6). Figure 2 demonstrates the distribution of reasons for not asking about QoL by health profession.

### 3.3. Ocular Allergy History Questions

**Figure 3:**
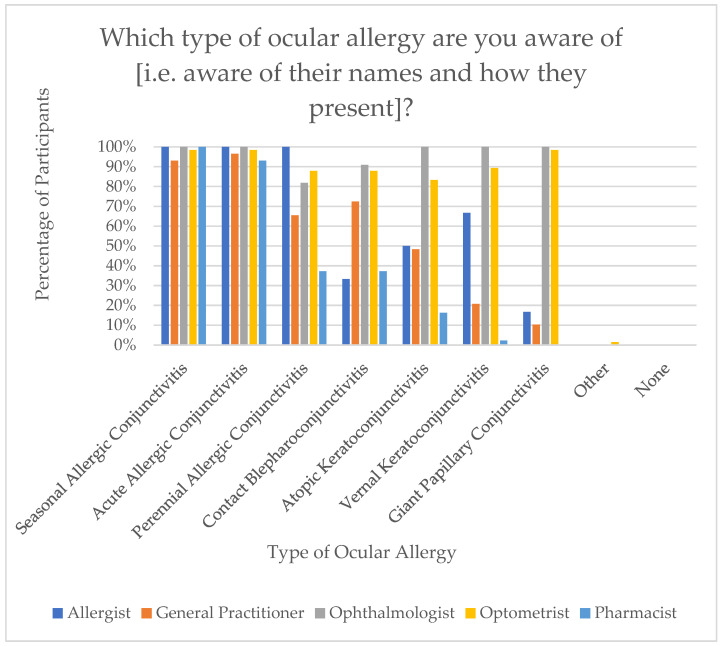
Awareness of different types of ocular allergy by health practitioner.

Participants’ awareness of various types of OA was assessed. The most recognised condition was Seasonal Allergic Conjunctivitis (SAC) (n = 152, 98.1%), followed by Acute Allergic Conjunctivitis (AAC) (n = 150, 96.8%), Perennial Allergic Conjunctivitis (PAC) (n = 108, 69.7%), Contact Blepharoconjunctivitis (CBC) (n = 107, 69.0%), Atopic Keratoconjunctivitis (AKC) (n = 90, 58.1%), Vernal Keratoconjunctivitis (VKC) (n = 81, 52.3%), then Giant Papillary Conjunctivitis (GPC) (n = 80, 51.6%). Notably, Ophthalmologists demonstrated the highest level of awareness across all OA types, while Pharmacists reported the lowest awareness levels. Figure 3 demonstrates this distribution.

Itchiness, recognised as the hallmark symptom of OA, was correctly identified by 83.2% of participants, whilst 16.8% were unaware that this was the hallmark symptom of OA. A comparison of practitioner awareness using Fisher’s exact test (*p* = 0.000), showed statistically significant differences among the professions. Optometrists had the highest awareness (96.9%), followed by General Practitioners (75.9%), Ophthalmologists (72.7%), Pharmacists (72.1%), and Allergists/Immunologists (66.7%).

**Figure 4:**
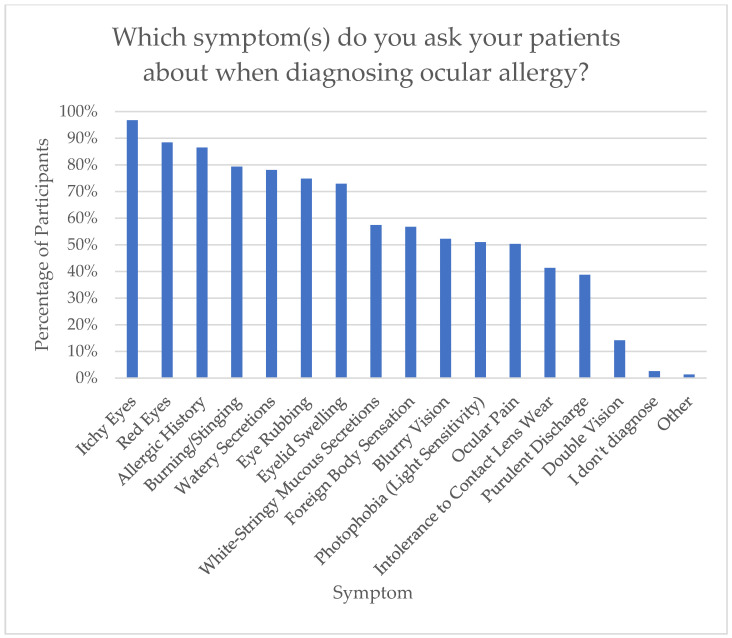
Symptoms asked in ocular allergy history taking.

An item to gauge which symptoms health practitioners ask about if they suspect an OA was implemented. The top symptom asked was itchy eyes (n = 150, 96.8%), followed by red eyes (n = 137, 88.4%), allergic history (n = 134, 86.5%), burning/stinging (n = 123, 79.4%), and watery secretions (n = 121, 78.1%). It is significant to note that eye rubbing was only asked by 74.8% of participants. This is demonstrated in Figure 4.

This correlated with the results found in the following item assessing how often the participant asked their patient about eye rubbing, which showed that only 39.4% of participants ‘always’ asked about eye rubbing, followed by frequently (27.1%), sometimes (19.3%), rarely (9.7%), and never (4.5%). Furthermore, a non-parametric analysis was performed (degrees of freedom = 23.009), which was statistically significant (*p* = 0.0001). It was found that the Allergists/Immunologists and Ophthalmologists median was Always, GPs and Optometrists median was Frequently, and Pharmacists median was Sometimes.

An item about how often practitioners communicate to participants not to rub their eyes was also implemented. It was found that 39.4% ‘always’ communicated to patients not to rub their eyes, followed by frequently (33.5%), sometimes (13.5%), rarely (8.4%), and never (5.2%). This demonstrated consistency with the above results. Furthermore, a non-parametric analysis was performed (degrees of freedom = 23.708), which was statistically significant (*p* = 0.0001). It was found that Ophthalmologists’ and Optometrists’ median was Always, Allergists/Immunologists’ median was Frequently–Always, GPs’ median was Frequently, and Pharmacists’ median was Sometimes.

### 3.4. Diagnostic Methods in Ocular Allergy

The diagnostic tools utilised in assessing OA varied across health practitioners. All Optometrists (100.0%) and Ophthalmologists (100.0%) utilised slit lamp assessment to detect signs of OA. Interestingly, a small percentage (13.8%) of GPs also used this. Likewise, most Ophthalmologists (90.9%) and Optometrists (95.5%) used the slit lamp for signs of differential diagnosis. Similarly, most Ophthalmologists (90.9%) and Optometrists (87.9%) used slit lamp for tear film assessment. Likewise, a small percentage of GPs also used slit lamp for differential diagnoses and tear film assessment at 10.3% and 6.9%, respectively. However, the most common diagnostic method amongst GPs (51.7%) was only asking about symptoms to diagnose OA patients, which was also seen with Pharmacists (67.4%). In contrast, 100% of Allergists/Immunologists primary relied on the skin prick test as a method of diagnosis. Additionally, both Allergists/Immunologists (83.3%) and GPs (13.8%) made use of serum IgE. Specialist diagnostic tests such as topography were only used by a small percentage of Ophthalmologists (18.18%) and Optometrists (24.24%). Similarly, tear film assessment with keratography was also used by a small percentage of Ophthalmologists (9.09%) and Optometrists (10.61%). Most health practitioners would refer at some point for diagnostic procedures to be implemented.

### 3.5. Management Methods in Ocular Allergy

**Figure 5:**
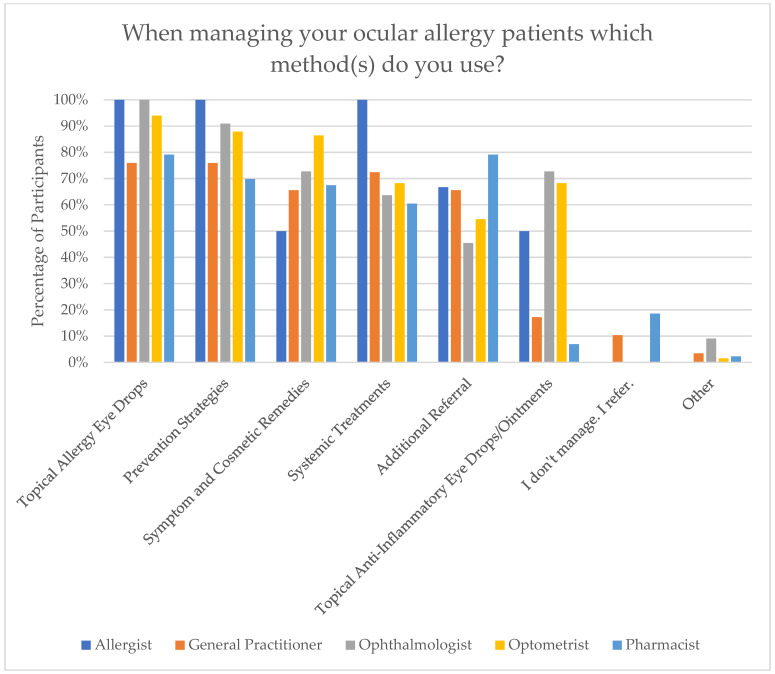
Management methods used in ocular allergy.

An item covering all management methods used in OA was implemented. The most used management method was topical allergy eye drops (n = 135, 87.1%), followed by prevention strategies (n = 126, 81.3%), symptom and cosmetic remedies (n = 116, 74.8%), systemic treatments (n = 105, 67.7%), additional referral (n = 98, 63.2%), topical anti-inflammatory eye drops/ointments (n = 64, 41.3%), and some chose not to manage and were only referred (n = 11, 7.1%). The top three management strategies employed by Allergists/Immunologists and GPs were prevention strategies, topical allergy eye drops, and systemic treatments. Among Ophthalmologists, the leading methods were topical allergy eye drops, prevention strategies, followed by symptom and cosmetic remedies and topical anti-inflammatory eye drops/ointments, used equally. Optometrists primarily relied on topical allergy eye drops, prevention strategies, and symptom and cosmetic remedies. Pharmacists most commonly managed OA through referrals and topical allergy eye drops, used equally, followed by prevention strategies, then symptom and cosmetic remedies. These results are demonstrated in Figure 5.

Participants were invited to specify the exact management methods they used. An interesting finding was the fact that health practitioners appeared unaware of active ingredients in topical allergy eye drops. For instance, while selecting antihistamine eye drops, they often cited a product containing both antihistamine and mast cell stabilisers. Another interesting finding was that 11.0% of practitioners prescribed vasoconstrictor eyedrops and 5.2% of practitioners prescribed and antihistamine–vasoconstrictor combination eyedrops. These are generally inappropriate due to prolonged effects [8,9]. Such drops should be reserved for reducing hyperaemia for cosmetic purposes, if other methods have been unsuccessful.

### 3.6. Knowledge on Ocular Allergy

This domain assessed health practitioners’ knowledge on OA. The first item aimed to understand which properties, apart from antihistamine and mast cell control, do some anti-allergy eye drops possess. The correct response was eosinophil inhibition [10,11,12]. Interestingly, 58.7% of participants were unsure and only 30.3% of participants achieved the correct response. The remaining participants selected basophil inhibition (5.2%), neutrophil inhibition (5.2%), or other (0.6%). Additionally, a comparison of each practitioner selecting the correct response was assessed using Fisher’s exact test (*p* = 0.181). However, this difference was not statistically significant.

Participants were also assessed on their understanding of the indications of use for mast cell stabiliser eye drops, with the correct response being Prophylaxis [13,14]. A total of 74.9% of participants selected the correct response, followed by unsure (11.6%), reduction of red eyes (10.3%), and other (3.2%). A comparative analysis was also conducted using Fisher’s exact test (*p* = 0.000), which was statistically significant. Notably, Optometrists (92.4%) had the highest rate of selecting the correct response followed closely by Ophthalmologists (90.91%), then Pharmacists (67.4%), Allergists/Immunologists (50.0%), and GPs (44.8%). This is presented in Table 4.

Another item in the survey aimed to assess participants’ understanding of the cause of itching in OA, where histamine was the correct response [15]. An amount of 67.7% of participants selected the correct response, followed by unsure (19.4%), IgE antibodies (11.6%), and T-cells and B-cells (1.3%). A comparative analysis was also conducted using Fisher’s exact test (*p* = 0.606). However, this was not statistically significant.

Three items regarding side effects and/or precautions of eye drops that may be used in OA were implemented. Firstly, participants were assessed on vasoconstrictors side effects and/or precautions. It was appropriate to select all responses [16,17]. However, no single response was selected by all participants. A total of 70.3% considered rebound hyperaemia with most of those being Optometrists (86.4%) and Ophthalmologists (81.8%), and least being GPs (44.83%). However, GPs strongly considered glaucoma (65.5%).

Secondly, participants were assessed on corticosteroids side effects and/or precautions. It was appropriate to select all responses [16,17]. However, no single response was selected by all participants. In total, 79.4% considered increased intraocular pressure, 60.65% considered risk of infection, and 58.65% considered cataracts.

Finally, participants were assessed on non-steroidal anti-inflammatory [NSAIDS] eye drop side effects and/or precautions. It was appropriate to select all responses [16,17]. However, no single response was selected by all participants. In fact, this item showed the most significant knowledge gap as all responses were only selected by 50% or less. Patterns in health practitioner groups showed strong knowledge among Ophthalmologists and weaker knowledge amongst GPs with 44.8% of them selecting ‘Unsure’.

An item assessing the use of calcineurin inhibitor use found that 94.2% of participants do not use calcineurin inhibitors for their OA patients. A comparative analysis was conducted using Fisher’s exact test (*p* = 0.001), which was statistically significant. Only Ophthalmologists, Optometrists, and Allergists/Immunologists used calcineurin inhibitors. For these health practitioners where calcineurin inhibitor use is within their scope of practice, the main reasons for not prescribing, which was found qualitatively, were lack of awareness of their benefits, not requiring them due to success with other treatments, lack of confidence in prescribing, and accessibility issues.

Participants were also assessed on how often they considered preservatives when prescribing to OA patients. Most participants selected always (38.1%), followed by frequently (32.2%), sometimes (22.6%), rarely (5.2%), and never (1.9%). Furthermore, a non-parametric analysis was performed (degrees of freedom = 8.850), which was statistically significant (*p* = 0.0433). It was found that Ophthalmologists’, Optometrists’, and Pharmacists’ median was Frequently, Allergists/Immunologists’ median was Sometimes–Frequently, and GPs’ median was Sometimes.

### 3.7. Collaborative Care in Ocular Allergy

The Collaborative Care in the OA domain was implemented to gain perspectives of health practitioners. Most participants (90.3%) indicated that they did refer to other health practitioners for their OA patients. However, 68.4% of participants said they did not believe there was a clear collaborative care model, 14.2% were unsure, and only 17.4% believed there was a clear collaborative care model. A comparative analysis was also conducted using Fisher’s exact test (*p* = 0.008), which was statistically significant. This showed that only 31.0% of GPs think there is a clear collaborative care model in Australia, followed by Pharmacists (23.3%), Allergists/Immunologists (16.7%), then Optometrists (10.6%) and Ophthalmologists (0%).

## 4. Discussion

This survey has proven beneficial in exploring Australian health practitioners’ awareness and implementation of evidence-based approaches in OA. It is significant to note that this survey is not representative of each specialty’s knowledge and practices, but rather is a preliminary study aimed at determining health practitioners’ awareness and implementation of diagnostic, treatment, and collaborative care approaches to OA.

### 4.1. Characteristics

The characteristics section was useful in understanding perceptions of health practitioners on their exposure to OA patients, current education on OA, and confidence levels on OA. The specialty of Pharmacists (4.7%) was the only one that had participants indicate they ‘never’ see OA patients. However, the current literature reports that 3% of patients presenting to pharmacies are OA sufferers [18]. This may be due to accessibility of medications where it was found that up to 73% of OA patients will use OTC medications [19]. Thus, this item served as a predictor for perhaps a lack of awareness that the patients presenting to pharmacy practices are OA patients, given that the patients are most likely only presenting to purchase OTC medications and the Pharmacists’ input was not sought. Therefore, lower implementation of diagnostic, treatment, and collaborative care approaches were seen amongst Pharmacists. Further to this, the highest rates of education was in Ophthalmologists and Optometrists, who received continuing education weekly, and the lowest rates of continuing education was amongst Pharmacists and GPs, who stated they never received continuing education on OA. This perhaps explains the confidence levels on OA, which revealed that Ophthalmologists and Optometrists had the highest confidence levels, whilst the lowest confidence levels were among the Pharmacists and GPs.

### 4.2. Quality of Life in Ocular Allergy Patients

The evaluation of QoL in patients with OA is a critical aspect of comprehensive patient care, yet the findings revealed that only 42.6% of practitioners reported inquiring about QoL [20]. This aligns with the previously mentioned studies which revealed that patient perceptions of the severity of their OA were contrary to practitioner evaluation [2,3].

Allergists/Immunologists had the highest rates of asking about QoL, while Pharmacists had the lowest. The difference may reflect the differing scopes of practice and patient interaction among these health professionals. For instance, Allergists/Immunologists are directly involved in managing allergy related symptoms, so they may prioritise investigating patients’ QoL, whereas a Pharmacist traditionally focuses on medication dispensing and education [21]. Furthermore, of the participants who did ask about QoL, only 4.5% of them used currently established validated questionnaires. Likewise, health practitioners who did not ask about QoL cited a lack of awareness regarding currently available questionnaires [20] as their primary reason for omission, with the highest rates of this reason being amongst Optometrists, Pharmacists, and GPs. This suggests that educational initiatives at improving knowledge about validated QoL measures could enhance practitioners utilising such validated questionnaires. However, several other reasons including not being essential and its time-consuming nature was also found amongst Optometrists and Ophthalmologists, revealing that appointment time-constraints may affect inclusion of QoL assessment in clinical routine. Overall, if QoL is not assessed, then this goes against the notion of patient-centered care, as it cannot be established whether a patient’s OA is improving. This is particularly significant as OA cannot be treated and only managed. Thus, symptom management is important in improving patients QoL, therefore making QoL an important measure to quantitatively ensure success of management.

### 4.3. Ocular Allergy History Questions

In the OA history-taking domain, it was interesting to find that only 83.2% of participants were aware that the hallmark symptom of OA is itchy eyes. This is perhaps the current clearest diagnostic measure of OA, which differentiates it from other presentations of red eye [22]. This was reiterated in the symptom questions asked, whereby asking about itchy eyes also fell short of 100%. Consequently, only 79.3% of participants asked about eye rubbing, which correlated with the following items assessing frequency of asking about eye rubbing and educating patients about avoiding eye rubbing. Specifically, Ophthalmologists and Allergists/Immunologists were those that mostly recognised the importance of eye rubbing whereas Pharmacists were those that inquired and educated the least about eye rubbing. It is significant to mention that Optometrists and GPs also did not ask about eye rubbing regularly; however, Optometrists regularly communicated the importance of avoiding eye rubbing whereas GPs did not. Thus, further education needs to be provided to *all* health practitioners, particularly Pharmacists, regarding the significance of itchy eyes as the hallmark feature of OA, and the importance of questioning about eye rubbing due to the negative effects and potential progression to keratoconus [23,24].

### 4.4. Diagnostic and Management Methods in Ocular Allergy

Through the results established in the diagnostic methods in OA and management methods in OA domains, there were low awareness and implementation of evidence-based practices. For instance, it was found that there was a lack of awareness of active ingredients in topical allergy eye drops. Not knowing the active ingredient may result in recommendation of inappropriate dosing, which may result in patients not responding to treatment [25]. A comparative study assessing the dose log and percentage inhibition of ketotifen and olopatadine found that ketotifen had a narrow therapeutic range where it is in positive inhibition. Ketotifen can stimulate, rather than prevent histamine release at concentrations that are slightly higher than effective inhibitor concentrations [25]. It is therefore essential to note that if Ketotifen is not given at a twice daily dosage and a higher dosage is given, then this may result in worsening symptoms. Thus, this highlights that practitioners need to have more awareness of the active ingredients in topical allergy eye drops.

### 4.5. Knowledge on Ocular Allergy

The knowledge on the OA domain found that health practitioners have a low understanding of mechanisms of OA and OA treatments. The first item revealed that only 30.3% of participants were aware that OA eye drops act on eosinophils [10,11,12]. This shows a low understanding of pathophysiological mechanisms of OA, which may aid in delivering targeted management methods. The following item was more promising having 78.8% of participants aware that mast cell stabilisers should be used prior to allergy season [13,14]; however, GPs had a lower understanding of this, revealing an area of education needs to be provided to ensure appropriate prescribing. This is essential since by stabilising the mast cells prior to allergy season, histamine release is avoided to ensure no itching occurs and thus, no eye rubbing. Moreover, it was found that only 67.7% of participants were aware that histamines cause itching, which is concerning given that histamine control is essential to avoid sequelae of the disease [23,24].

It was essential to also assess other eye drops that may be used in OA, apart from OA specific drops. Evidently, all health practitioners had a low awareness about the side effects and/or precautions of corticosteroid drops, vasoconstrictor drops, and NSAID drops in descending order, from most awareness to least awareness as per the distribution of numbers. This requires more education as these may result in inappropriate prescribing. Furthermore, calcineurin inhibitors was only used by 5.8% of participants. This is concerning as the VEKTIS study suggests the effectiveness of calcineurin inhibitors in OA [26]. Thus, further education needs to be provided in this area, particularly to increase prescribing confidence. Finally, it was found that Allergists/Immunologists had a low consideration of preservatives in eye drops, and Ophthalmologists and Optometrists had the highest consideration. Therefore, education surrounding the side-effects of preservatives is necessary [27].

### 4.6. Collaborative Care in Ocular Allergy

The collaborative care domain gained perspectives on collaborative care on OA in Australia. Although most participants (90.3%) referred their OA patients, most participants did not believe there was a collaborative care model, whilst some were unsure. This could cause an increased burden in the healthcare system through longer wait times and increased costs. Thus, a collaborative care model for OA in Australia needs to be determined and should be evaluated in future studies. This has already been established for other ocular conditions and has proven successful in reducing time and costs associated with the burden of the disease [28].

### 4.7. Limitations

A limitation of the study was the small number of Allergists/Immunologists and Ophthalmologists involved than the anticipated sample size, even though all recruitment methods were exhausted. However, this was expected given the time commitments of such health practitioners, and the number of these health practitioners in Australia. Nonetheless, it was still possible to draw on awareness and implementation in the diagnostic, treatment, and collaborative care approaches of health practitioners on OA, as a preliminary study. In future, this study should be conducted on a larger scale to assess all health practitioner groups’ knowledge and practices.

Another limitation was that the prescribing patterns were difficult to be gauged (i.e., dosages and reasons for prescribing). Future studies should implement case scenarios to understand this further.

Finally, all other limitations were primarily that a survey with closed ended questions may result in response bias as participants may not have mentioned certain options in an open-ended scenario.

## 5. Conclusions

The SOAHP proved beneficial in understanding current awareness and implementation of diagnostic, treatment, and collaborative care approaches regarding OA in Australian health practitioners. Overall, eye care practitioners including Ophthalmologists and Optometrists had higher awareness of presentations and management of OA; however, there remained gaps in adherence of evidence-based practices. Furthermore, Allergists/Immunologists also had a sound understanding of the OA as subset of the broader disease of allergy but likewise showed gaps in certain aspects of OA. Finally, GPs and Pharmacists had lower awareness and implementation rates of currently available evidence-based approaches in OA. However, these distinct patterns when comparing health practitioners are likely influenced by differences in training and clinical responsibilities. Yet, it is still significant that there is an overall decreased understanding surrounding evidence-based practices in OA to all health practitioners. This study should be carried out on a larger scale whereby practitioner groups are not compared against each other but rather aligned to a standard of care according to their scope of practice. Through this, further education can be provided to increase knowledge surrounding OA, and thus, better practices employed.

## Figures and Tables

**Table 1:** Demographic characteristics of survey participants (n = 155).

Characteristic	All, n (%)
*Sex*	
Male	58 (37.4)
Female	97 (62.6)
*Age (Years)*	
Range	21–70
Mean	36.2 ± 12.0
*Years of Practice*	
Range	1–48
Mean	10.0 ± 10.1
*Career Stage*	
Early Career (<3 Years)	48 (31)
Early–Mid Career (3–5 Years)	32 (20)
Mid–Late Career (5–12 Years)	37 (24)
Late Career (>12 Years)	38 (25)
*State*	
New South Wales	81 (52.3)
Queensland	15 (9.7)
South Australia	5 (3.2)
Tasmania	3 (1.9)
Victoria	33 (21.3)
Western Australia	16 (10.3)
Undetermined	2 (1.3)
*Modality of Practice*	
Full Time	95 (61.3)
Part Time	47 (30.3)
Locum/Casual	12 (7.7)
Other	1 (0.7)
*Place of Practice*	
Group Practice	85 (54.8)
Hospital	32 (20.7)
Solo/Individual Practice	25 (16.1)
Community Health Centre	3 (1.9)
Other	10 (6.5)

**Table 2:** Demographic characteristics of survey participants by profession.

	Allergist, n (%)	General Practitioner, n (%)	Ophthalmologist, n (%)	Optometrist, n (%)	Pharmacist, n (%)
*Sex*					
Male	3 (50.0)	8 (27.6)	10 (90.9)	29 (43.9)	8 (18.6)
Female	3 (50.0)	21 (72.4)	1 (9.1)	37 (56.1)	35 (81.4)
*Modality of Practice*					
Full Time	2 (33.3)	13 (44.8)	10 (90.9)	39 (59.1)	31 (72.1)
Part Time	3 (50.0)	16 (55.2)	1 (9.1)	18 (27.3)	9 (20.9)
Locum/Casual	0 (0.0)	0 (0.0)	0 (0.0)	9 (13.6)	3 (7.0)
Other	1 (16.7)	0 (0.0)	0 (0.0)	0 (0.0)	0 (0.0)
*Place of Practice*					
Group Practice	0 (0.0)	24 (82.8)	7 (63.6)	43 (65.1)	11 (25.6)
Hospital	6 (100.0)	1 (3.4)	1 (9.1)	2 (3.0)	22 (51.1)
Solo/Individual Practice	0 (0.0)	2 (6.9)	3 (27.3)	17 (25.8)	3 (7.0)
Community Health Centre	0 (0.0)	0 (0.0)	0 (0.0)	0 (0.0)	3 (7.0)
Other	0 (0.0)	2 (6.9)	0 (0.0)	4 (6.1)	4 (9.3)

**Table 3:** Asking ocular allergy patients about quality-of-life comparative analysis by health practitioner (*p* = 0.024).

	Allergist, n (%)	General Practitioner, n (%)	Ophthalmologist, n (%)	Optometrist, n (%)	Pharmacist, n (%)	Total, n (%)
Yes	4 (66.7)	10 (34.5)	6 (54.6)	35 (53.0)	11 (25.6)	66 (42.6)
No	2 (33.3)	19 (65.5)	5 (45.5)	31 (47.0)	32 (74.4)	89 (57.4)
Total	6 (100.0)	29 (100.0)	11 (100.0)	66 (100.0)	43 (100.0)	155 (100.0)

**Table 4:** Correct indications for mast cell stabiliser comparative analysis by health practitioner (*p* = 0.000).

	Allergist, n (%)	General Practitioner, n (%)	Ophthalmologist, n (%)	Optometrist, n (%)	Pharmacist, n (%)	Total n (%)
Prophylaxis	3 (50.0)	13 (44.8)	10 (90.9)	61 (92.4)	29 (67.4)	116 (74.8)
Other	3 (50.0)	16 (55.2)	1 (9.1)	5 (7.6)	14 (32.6)	39 (25.2)
Total	6 (100.0)	29 (100.0)	11 (100.0)	66 (100.0)	43 (100.0)	155 (100.0)

## Data Availability

The data associated with this manuscript are available from the corresponding author upon reasonable request.

## Abbreviations

The following abbreviations are used in this manuscript:

AAC	Acute Allergic Conjunctivitis
AHPRA	Australian Health Practitioner Regulation Agency
AKC	Atopic Keratoconjunctivitis
AMA	Australian Medical Association
ASCIA	Australasian and New Zealand Society of Immunology
ASI	Australian and New Zealand Society of Immunology
CBC	Contact Blepharoconjunctivitis
EAPIQ	Eye Allergy Patient Impact Questionnaire
GPC	Giant Papillary Conjunctivitis
GPs	General Practitioners
IQR	Interquartile Range
NSAID	Non-steroidal Anti-Inflammatory Drugs
OA	Ocular Allergy
OTC	Over-the-Counter
PAC	Perennial Allergic Conjunctivitis
QoL	Quality of Life
RACGP	Royal Australian College of General Practitioners
SAC	Seasonal Allergic Conjunctivitis
SOAHP	Survey on Ocular Allergy for Health Practitioners
USA	United States of America
VKC	Vernal Keratoconjunctivitis
